# Dynamic Cooperative Clustering Based Power Assignment: Network Capacity and Lifetime Efficient Topology Control in Cooperative Ad Hoc Networks

**DOI:** 10.1155/2014/219210

**Published:** 2014-03-16

**Authors:** Xiao-Hong Li, Ling Xiao, Dong Wang

**Affiliations:** College of Information Science and Engineering, Hunan University, Changsha 410082, China

## Abstract

Cooperative communication (CC) is used in topology control as it can reduce the transmission power and expand the transmission range. However, all previous research on topology control under the CC model focused on maintaining network connectivity and minimizing the total energy consumption, which would lead to low network capacity, transmission interruption, or even network paralysis. Meanwhile, without considering the balance of energy consumption in the network, it would reduce the network lifetime and greatly affect the network performance. This paper tries to solve the above problems existing in the research on topology control under the CC model by proposing a power assignment (DCCPA) algorithm based on dynamic cooperative clustering in cooperative ad hoc networks. The new algorithm clusters the network to maximize network capacity and makes the clusters communicate with each other by CC. To reduce the number of redundant links between clusters, we design a static clustering method by using Kruskal algorithm. To maximize the network lifetime, we also propose a cluster head rotating method which can reach a good tradeoff between residual energy and distance for the cluster head reselection. Experimental results show that DCCPA can improve 80% network capacity with Cooperative Bridges algorithm; meanwhile, it can improve 20% network lifetime.

## 1. Introduction

Wireless ad hoc networks consist of wireless nodes that can communicate with one another in the absence of a fixed infrastructure. There are three important issues in wireless ad hoc networks. The first is network connectivity. The network is connected if any two nodes can communicate with each other in a network. It is much more difficult to ensure that two nodes connect in a wireless network than in a wired network because of the instable wireless channel, signal attenuation, and so on. Network connectivity is closely related to the node transmission power. The greater the node transmission power, the better the network connectivity [1]. The second is energy consumption. Wireless nodes have a limited operational time as they are battery powered [2]. It is nearly impossible for the battery at nodes to be recharged or replaced; thus, energy efficiency is crucial in wireless ad hoc networks. The key difference between wireless ad hoc networks and conventional communication structures, from the designer's point of view, lies in the power assignment model [3]. The energy consumption of a single node is proportional to the transmission power it assigned. The greater transmission radius of a node means that it requires higher transmission power. We evaluate the energy efficiency by two parameters: total energy consumption and network lifetime. The third is network capacity which is simply defined as the amount of data transmitted in a certain period of time. Future mobile wireless communication networks must be able to bear high-speed, highly efficient business processes if it is to support multimedia business featured by real-time and a large amount of data. Two main factors affect the network capacity: the transmission power and the radio interference caused by simultaneous transmission. According to typical Shannon theorem, the greater the transmission power, the greater the network capacity [4]. That is, there is a compromised relationship between reducing energy consumption and increasing network capacity.

Topology control is one of the most important techniques used in wireless ad hoc networks to reduce energy consumption or increase network capacity on the premise of maintaining connectivity by adjusting the transmission power [1]. However, the actual wireless network is difficult to be fully connected initially, making the topology control to operate poorly, so some researchers introduce cooperative communication to apply in topology control to solve this problem [5].

**Figure 1:**
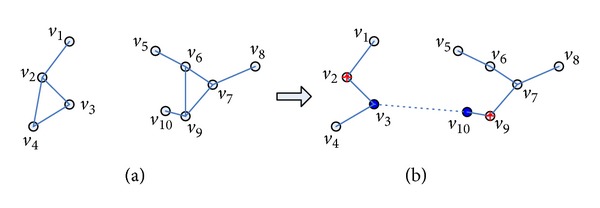
Interference in a sparse network (resulting topology graph in Cooperative Bridges).

Cooperative communication is a new wireless communication technology which allows single-antenna terminal devices to share their physical resources to communicate in a multiuser environment. It forms a virtual antenna array by utilizing the broadcast feature of a signal [6, 7], which achieves resource sharing and expands the coverage of network through mutual cooperation [8]. It is called cooperative ad hoc networks after using cooperative communication in wireless ad hoc networks. Energy consumption and network capacity issues are still very important in cooperative ad hoc networks. The previous works [2, 5, 9] mainly study the topology control problem in cooperative ad hoc networks to minimize the energy consumption and guarantee the network connectivity by constructing a sparse graph. They ignore the network capacity efficiency because they do not consider reducing the interference in a sparse graph, which will affect the network capacity destructively and eventually reduce the network capacity [10].  Figure 1 shows an example. At first the initial network is disconnected, as Figure 1(a) shows. Then CC in [5] is applied to make network connected; *v*_3_ *v*_10_ is a CC link and the final resulting topology structure is similar to the MST, which is a sparse graph and can reduce interference to a certain extent, but neighbors will still interfere with each other when nodes transmit simultaneously. As Figure 1(b) shows, when node *v*_6_ transmits a message to node *v*_7_, nodes *v*_8_ and *v*_9_ will cause interference to *v*_7_; when node *v*_7_ transmits a message to *v*_6_, node *v*_5_ will cause interference to *v*_6_. So, the previous works of topology control problem in cooperative ad hoc networks may not work efficiently in terms of network capacity.

**Figure 2:**
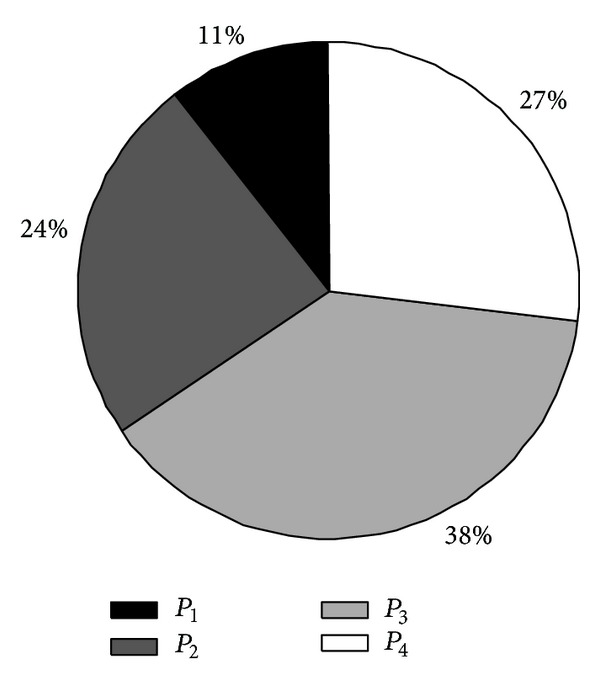
The average node energy consumption distribution.

**Figure 3:**
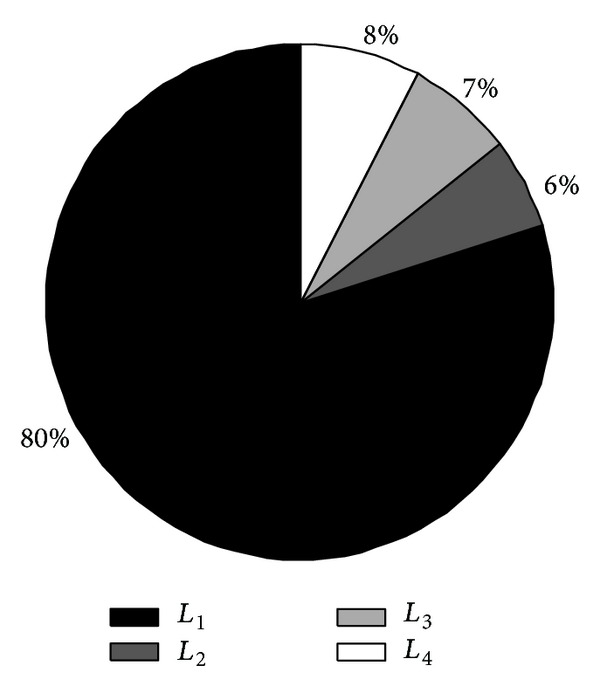
The distribution of the remaining energy of nodes when the first node dies.

Apart from ignoring the network capacity efficiency, previous research on topology control under the CC model failed to equalize the energy consumption among network nodes. Assume that the maximal transmission power of each node is *P*_max⁡_, *P*_1_ = (75%*P*_max⁡_, *P*_max⁡_]; *P*_2_ = (50%*P*_max⁡_, 75%*P*_max⁡_]; *P*_3_ = (25%*P*_max⁡_, 50%*P*_max⁡_]; *P*_4_ = [0%*P*_max⁡_, 25%*P*_max⁡_].  Figure 2 shows the average node energy consumption distribution of 100 topologies (suppose there are 100 nodes) built by Cooperative Bridges algorithm [5]. In the interval *P*_1_ there are only 11% nodes, which transmit messages in a relatively high transmission power. However, according to this algorithm, the network topology structure is static and immutable during the whole network lifetime. These will lead to the fact that nodes in the *P*_1_ transmit messages at a higher transmission power, thus suffering faster energy consumption, while nodes in other intervals transmit messages at a lower transmission power, and the energy consumption rate is slower accordingly. This means that when those few nodes which consume much energy die earlier, most of the nodes which consume little energy still have much residual energy. This situation is visually shown in Figure 3. Assume the primary energy of each node is *E*, *L*_1_ = (75%*E*, *E*]; *L*_2_ = (50%*E*, 75%*E*]; *L*_3_ = (25%*E*, 50%*E*]; *L*_4_ = [0%*E*, 25%*E*]. Figure 3 shows the distribution of the remaining energy of nodes when the topology structure constructed by random network in Figure 2 comes to a final death. We can see from Figure 3 that, when the first node dies, the majority nodes of 80% stay in the high residual energy level L1. So, the uneven node energy consumption has a serious impact on network lifetime.

Therefore, the paper attempts to study the problem of how to maximize network capacity and energy efficiency in cooperative ad hoc networks. We design a topology control algorithm, called DCCPA, based on a dynamic cooperative cluster algorithm and power assignment to maximize its network capacity and network lifetime.

The paper is organized as follows. We summarize some related work in Section 2. Then we present the network model, introduce some definitions and statements which we use throughout the paper, and give a problem definition in Section 3.  Section 4 studies our topology algorithm. Afterwards we present some simulation results in Section 5. Finally, we draw some conclusion in Section 6.

## 2. Related Work

In this section, we focus on related works about topology control problems in cooperative ad hoc networks.

The main goal of designing cooperative ad hoc networks is to increase network capacity, reduce energy consumption, and enhance network coverage. What deserves our attention is that there is a tradeoff between the three. The way of choosing relay nodes is decided by the needs of different systems which choose different optimized objects for optimization. It is a hot issue to determine the number of relay nodes in a relay node selection algorithm.

As topology control is a network layer issue, researchers usually consider simple physical characteristics instead of variations of channel state when CC is applied in topology control. In [11], Guan et al. abstracted the topology control problem in cooperative ad hoc networks for a discrete stochastic optimization problem and they viewed network capacity as the goal of optimization. However, the authors consider that the time-varying channels have important impact on the network capacity. In [2], Cardei et al. first studied the topology control problem under cooperative model (denoted by TCC) which aims to obtain a strongly connected topology with minimum total energy consumption. In [9], Zhu et al. proposed two topology control algorithms to build cooperative energy spanners in which the energy efficiency of individual paths is guaranteed. In [5], Yu et al. applied CC model in topology control to improve the network connectivity as well as reducing transmission power. Their algorithm first constructs all candidates of bidirectional links by using CC model which can connect different disconnected components. Then they apply a 2-layer MST structure (one MST over the CC links and the other is inside each component) to further reduce the energy consumption. This is the best work in minimizing the total energy consumption while increasing the network connectivity. However, the proposed algorithm decreases the performance of network capacity greatly, which is validated by the experimental results. Therefore, the problem that the sparseness of the constructed topology under CC model should make network capacity and energy efficient at the same time is critical.

Such problem has been studied in traditional topology control algorithm (without CC) [3]. In [3], Shpungin and Li developed a static cluster based power assignment algorithm which achieves energy efficiency and also maximizes network capacity in the case of unlimited maximum transmission power of each node. Cluster algorithm is a kind of smart scheduling algorithms to improve the network capacity by lowering the interference of concurrent transmissions in a sparse network [12, 13]. In cluster algorithm, nodes are assigned transmission time slots so that only a subset of nodes are active at any given time slot while the others are idle. This scheme requires a synchronization mechanism, such as TDMA. In clustering algorithm, the energy consumption of cluster head is generally much higher than the cluster member nodes. Static clustering algorithm can easily lead to an early death of the cluster head node due to energy depletion. To avoid this situation, the cluster head rotation mechanism [14–16] is adopted so that each node serves as the cluster head in turn, ensuring that the residual energy of each node is equal to one another as close as possible. Cluster head rotation mechanism is usually independent of the clustering algorithm; they are complementary with each other instead. However, so far there is no network capacity and network lifetime efficiency topology control algorithm proposed under CC model yet.

## 3. The Definition of Model and Problem

In this section, we describe a cooperative communication model and a network model for our topology control mechanism. In addition, we also introduce a topology control problem for network capacity and energy efficiency in cooperative ad hoc networks.

### 3.1. Cooperative Model

**Figure 4:**
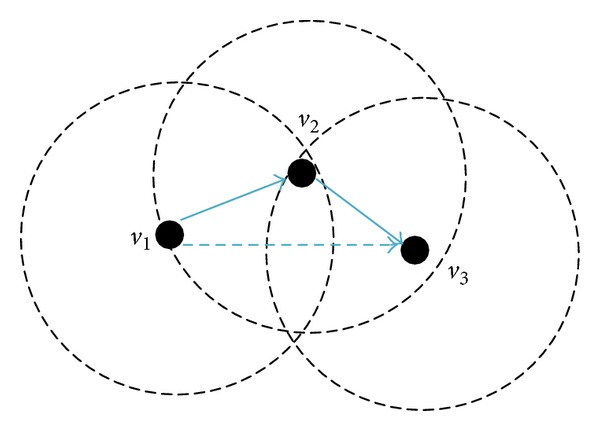
Simple CC model.

The simple explanation of the cooperative communication is given by a three-node example as shown in Figure 4. In the graph, node *v*_1_ is the source node, node *v*_3_ is the destination node, and node *v*_2_ is a relay node. The transmission from node *v*_1_ to node *v*_3_ is based on the frame-by-frame scheme. There are two time slots for a frame. In the first slot, node *v*_1_ makes a transmission to node *v*_3_. Because of the wireless broadcast feature, it is also overheard by relay node *v*_2_. In the second time slot, node *v*_2_ forwards the data received in the first time slot to node *v*_3_.

**Figure 5:**
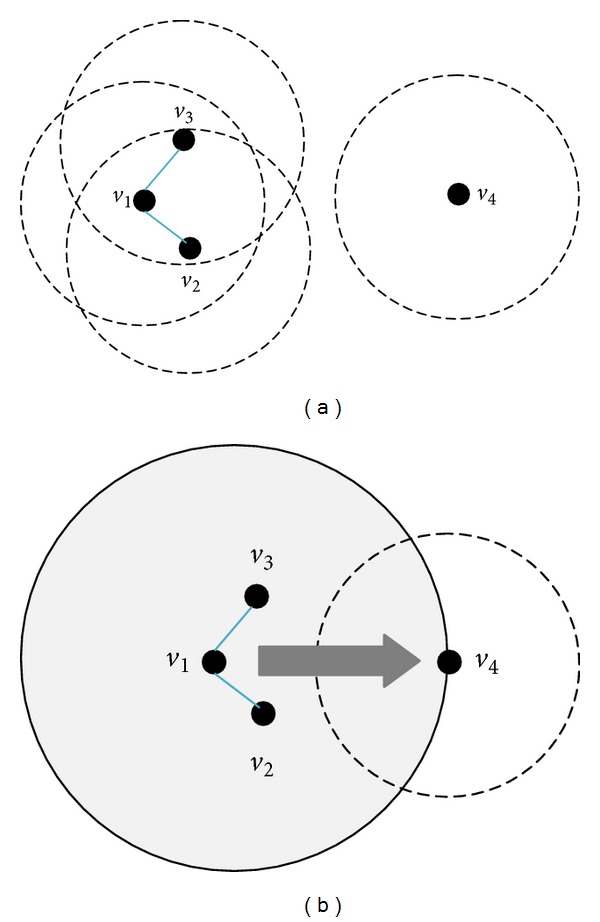
The second CC model.

There are two CC models in the paper. As Figure 4 shows, source nodes cannot communicate with target nodes directly while source nodes can communicate with relay nodes and relay nodes can directly communicate with target nodes. In this case, we look on the relay nodes as the help nodes of source nodes. This CC model is similar to [17, 18]. The other model which is showed in Figure 5 is that neither source nodes nor its neighbor nodes are able to communicate with destination nodes. The CC model is similar to [2, 5]. It takes the advantage of the physical layer design [14] that combines partial signals containing the same information to obtain the complete information.

Assume that each node has a maximum transmission power *P*_max⁡_. In the direct communication, that is, in the absence of cooperative communication, if a source node *v*_*i*_ can communicate with a destination node *v*_*j*_, they must satisfy

(1) $$\begin{array}{c} P_{i}{\left(d_{ij}\right)}^{-\kappa }\geq \tau \;\left(P_{i}\leq P_{\max}\right), \end{array}$$

where *P*_*i*_ is the transmission power of node *v*_*i*_ and *d*_*ij*_ is the Euclidean distance between *v*_*i*_ and *v*_*j*_, where *κ* is a constant representing the distance-power gradient, usually taken to be in the interval [1, 2, 4]. *τ* is the minimum average signal-to-noise ratio (SNR) for decoding received data.

When the first CC model is put to use, the transmission power of source node *v*_*i*_ and relay node *v*_*k*_ must meet the conditions below, respectively:

(2) $$\begin{array}{c} P_{i}{\left(d_{ik}\right)}^{-\kappa }\geq \tau \;\left(P_{i}\leq P_{\max}\right),\\ P_{k}{\left(d_{kj}\right)}^{-\kappa }\geq \tau \;\left(P_{i}\leq P_{\max}\right). \end{array}$$

When the second CC model is applied, if *v*_*i*_ transits the same signal with a set of help nodes *H*_*i*_, their transmission power satisfies

(3) $$\begin{array}{c} \sum_{v_{k}\in v_{i}\cup H_{i}}P_{k}{\left(d_{kj}\right)}^{-\kappa }\geq \tau \;\left(P_{i}\leq P_{\max}\right). \end{array}$$

### 3.2. Network Model

We consider a wireless ad hoc network with *n* nodes which are capable of receiving and combining partial received packets in accordance with the CC model. Every node *v*_*i*_ can adjust its transmission power *P*_*i*_ which is limited by a maximum value *P*_max⁡_. The network topology is modeled as a 2-dimensional directed graph: *G* = (*V*, *E*), where *V* = (*v*_1_,…, *v*_*n*_) denotes the set of wireless nodes and *E* denotes a set of directed communication links. We assume that each node has a unique ID and knows its own location information. Node ID and location information are exchanged among all nodes.

In this paper, we assume that a source node can choose one or more relay nodes to transmit message. Helper node set, helper link, node connectivity, and network connectivity's definitions are the same as [5]. Besides, we formally give some other definitions.

**Definition 1 (link capacity Cap(*v*_*i*_, *v*_*j*_))**

It is the maximum amount of messages that node *v*_*i*_ transmits to node *v*_*j*_ in unit time via wireless channel (*v*_*i*_, *v*_*j*_), that is,

(4) Cap(vi,vj)=Blog(1+SINR(vi,vj))=Blog(1+Pi/dijκN0+∑vk∈V∖{vi,vj}Pk/dkjκ),

where *B* is the channel bandwidth, SINR (*v*_*i*_, *v*_*j*_) is signal to interference plus noise ratio, and *N*_0_ is the ambient noise power, which is negligible comparing to the interference caused by other transmitting nodes.

A closer look at expression (4) reveals that the link capacity has a relationship with transmission power and radio interference. We denote *I*(*v*_*i*_, *v*_*j*_) = ∑_*v*_*k*_∈*V*∖{*v*_*i*_,*v*_*j*_}_ *P*_*k*_/*d*_*kj*_^*κ*^; *v*_*k*_ ∈ *V*∖{*v*_*i*_, *v*_*j*_} is the subset of nodes simultaneously transmitting at some time instant over a certain subchannel. Most of the paper is dedicated to analysis of how to reduce *I*(*v*_*i*_, *v*_*j*_), that is, radio interference.

**Definition 2 (path capacity Cap(*R*))**

It is the minimum channel capacity on the path *R*. The capacity of a path *R* in a communication graph *G* is defined as the capacity of the minimum capacity link in *R*, that is,

(5) $$\begin{array}{c} \text{Cap}\left(R\right)=\underset{\left(v_{i},v_{j}\right)\in R}{\min}\text{Cap}\left(v_{i},v_{j}\right). \end{array}$$

**Definition 3 (network capacity Cap(*G*))**

It is the minimum path capacity of all paths that *v*_*i*_ and *v*_*j*_ pass through, that is,

(6) $$\begin{array}{c} \text{Cap}\left(G\right)=\underset{v_{i},v_{j}\in V}{\min}\text{Cap}\left(R\right). \end{array}$$

**Definition 4 (network lifetime *L*(*G*))**

It is the time which takes the first node to run out of its battery charge.

### 3.3. Problem Formulation

Now we can define the new topology control problem, network capacity and energy efficient topology control in cooperative ad hoc networks (CEETC). Given a 2-dimensional directed graph *G*, which is strongly connected under CC model, assign transmission power *P*_*i*_ to every node *v*_*i*_ such that to (1) maximize network capacity Cap (*G*) and (2) maximize network lifetime *L*(*G*).

As TCC problem [2], which only maintains the connectivity and reduces energy consumption, has been proved to be NP-complete, it is a simple case of CEETC in cooperative ad hoc networks when ignoring capacity, so CEETC is also NP-complete. Therefore, we will propose a heuristic algorithm and give an approximate solution to CEETC problem.

## 4. Proposed Algorithm

In this section, we propose a dynamic cooperative clustering based power assignment (DCCPA) algorithm to solve the problem mentioned above. To keep the proposed algorithms simple and efficient, we only consider its one-hop neighbors as possible helper nodes for each node when CC is used [5].

This algorithm is divided into two steps: the first step is static clustering, including the intercluster communication and the transmission power assignment; the second step is cluster head rotating, including the cluster head election, the communication between cluster heads, and the transmission power reallocation. Before describing the algorithm, the pretreatment is conducted.

**Figure 6:**
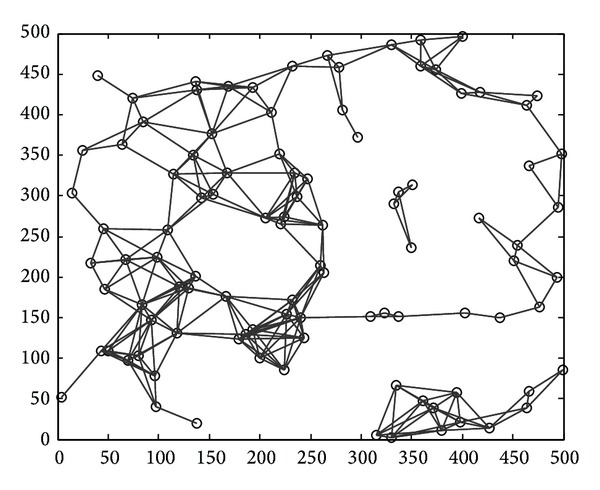
Disconnected networks.

We denote the wireless multihop network as an undirected simple graph *G* = (*V*, *E*), where *V* is the set of nodes; direct communication link and CC link are expressed by the solid and dotted lines, respectively. In graph *G*, first, *V*(*G*) ≠ *∅* and *E*(*G*) = *∅*. Then each node works with maximum power *P*_max⁡_, and from formula (1), the transmission radius of each node is $\sqrt{P_{\max}}$. If a direct path exists between the nodes *u*, *v*, edge (*u*, *v*) is constructed. When the network *G* is not connected, there are at least two groups after the construction of all edges has been completed, as Figure 6 shows. We describe every step in detail now.

**Figure 7:**
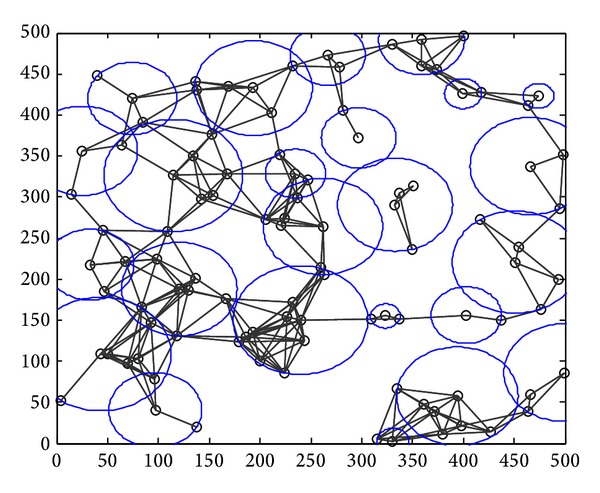
Clustering in step 2.

**Figure 8:**
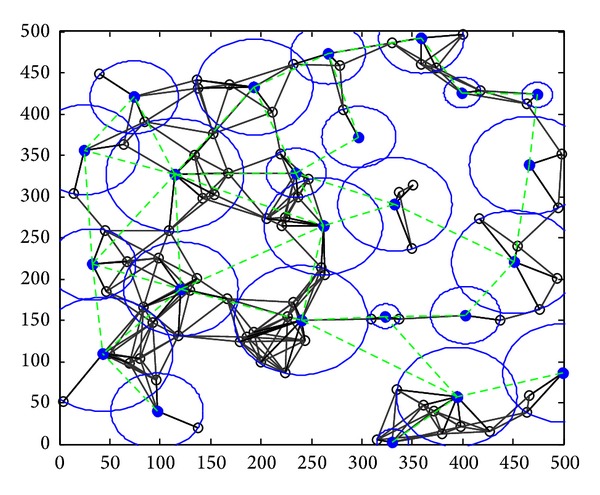
Redundant CC links among clusters in step 3.

**Figure 9:**
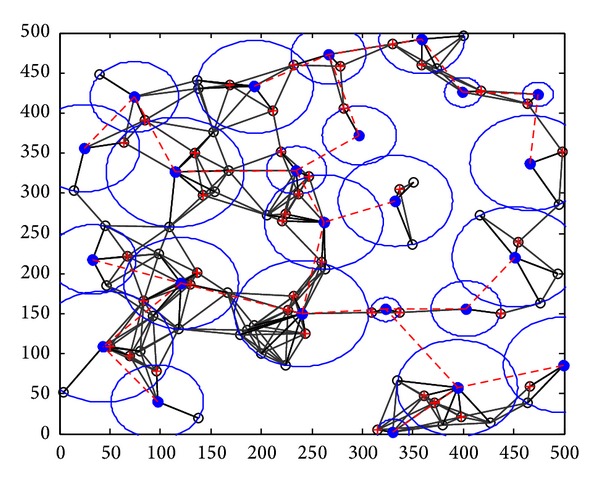
Minimum spanning tree regarding clusters as nodes.

**Step 1 (static clustering)**

(*1) Clustering in Graph G*. The clustering process is repeated. When clustering is completed, each node should belong to a cluster, either a cluster head or a cluster member. We use a set *T*(*G*) which is initialized to the set of all nodes; that is, *T*(*G*) = *V*(*G*). If *T*(*G*) is not empty, we select a node arbitrarily *u* ∈ *T*(*G*) to work as a cluster head. The cluster took shape from *u* and all the nodes which are with a distance of $\sqrt{P_{\max}}$ from *u*. The nodes in *M* are defined as the cluster members of *u*. Then we judge each node *v* ∈ *M* whether it belongs to another cluster. If not, *v* chooses *u* as its cluster. Otherwise, *v* chooses to be the cluster member with the nearest cluster head. Then we delete node *u* and its cluster members from *T*(*G*) and judge whether it is empty. If it is, clustering is completed. Otherwise, the process continues. The clustering process is shown in Figure 7; a circle represents a cluster.

(*2) Communicating between the Cluster Heads.* In this step, to make clusters communicate with each other, the way of selecting help nodes is the same as that of Greedy Heuristic in [5]. The process is shown in Figure 8. After CC links are established between clusters, Kruskal algorithm is used to derive its spanning tree (MST), as Figure 9 shows. Through the step, we can know helper nodes set *H*_*u*_ that each cluster head node *u* needs, and we can get *P*_*u*∪*H*_*u*__^*c*^(*v*) according to formula (3), which is the transmission power of *u* required to construct a CC link to the other cluster head node *v* with the help of helper nodes in *H*_*u*_:

(7) $$\begin{array}{c} P_{u\cup H\left(u\right)}^{c}\left(v\right)=\frac{\tau }{\sum_{i\in u\cup H(u)}{\left(d_{iv}\right)}^{-\kappa }}. \end{array}$$

(*3) Doing Power Assignment.* For the cluster members, what is needed is the energy which enables them to communicate with the cluster head they belong to; that is, for a cluster member *u*_*m*_, we assume its cluster head is *u* and the power of *u*_*m*_ is *d*_*uu*_*m*__^2^; that is,

(8) $$\begin{array}{c} p_{u_{m}}=\tau d_{uu_{m}}^{\kappa }. \end{array}$$

Because of the characteristics of helper nodes and cluster heads, it is relatively complex to assign power to them. For helper nodes, also cluster members, they should be able to help when they communicate with cluster heads they belong to. The power of each helper node *u*_*h*_ is decided by the following equation, where *u* is its cluster head and *v* is another neighbor cluster head:

(9) $$\begin{array}{c} P_{u_{h}}=\max\left\{d_{uu_{h}}^{2},\underset{\left(u,v\right)\in E\left(G^{\prime \prime }\right)}{\max}P_{u\cup H\left(u\right)}^{c}\left(v\right)\right\}. \end{array}$$

For the cluster head node, it not only should be able to communicate with each cluster member but also can communicate with its neighboring cluster heads; therefore the energy value assigned to cluster heads should be the maximum among these values. The power of each cluster head *u* is decided by the following equation, where *u*_*m*_ is its cluster member and *v* is another neighbor cluster head:

(10) $$\begin{array}{c} P_{u}=\max\left\{\underset{u_{m}\in M\left(u\right)}{\max}d_{uu_{m}}^{2},\underset{\left(u,v\right)\in E\left(G^{\prime \prime }\right)}{\max}P_{u\cup H\left(u\right)}^{c}\left(v\right)\right\}. \end{array}$$

**Figure 10:**
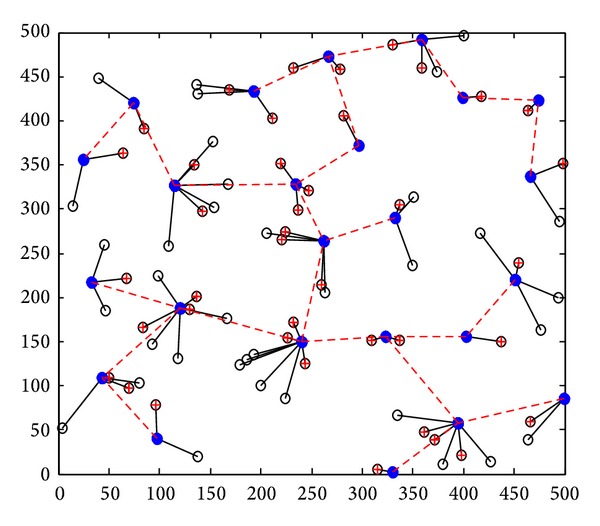
Final topology in step 4.

**Figure 11:**
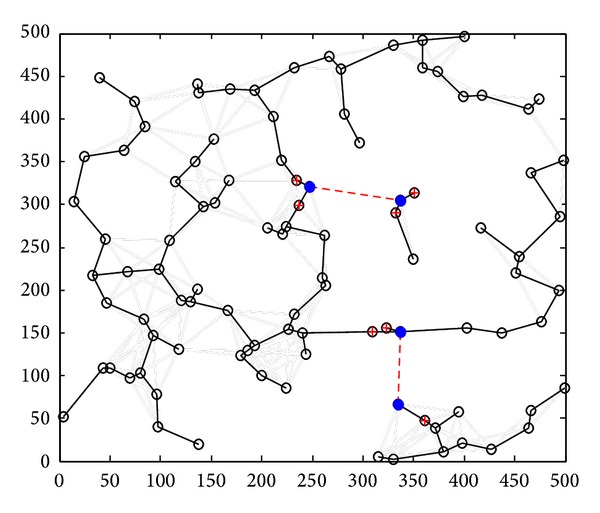
Final topology in Cooperative Bridges.

The final topology structure is shown in Figure 10, and the final topology structure in [5] is shown in Figure 11.

**Step 2 (cluster head rotating)**

(*1) Cluster Heads Reselection.* In the algorithm, the cluster head is not generated periodically; a passive changing manner is adopted instead. Namely, when the residual energy of a cluster head is less than the threshold value, a new cluster head is reselected dynamically. Assume that *v*_*i*_ is the other nodes in the current cluster that need to change cluster head node and *v*_*j*_ is the next-hop nearest cluster head node on the routing. The rule of reselecting a new cluster head is as follows: we always reselect the node of the largest *R*_*i*_ which stands for the tradeoff between residual energy *E*_*i*_ and distance *d*_*ij*_. *R*_*i*_ can be obtained by the following:

(11) $$\begin{array}{c} R_{i}=\frac{E_{i}}{d_{ij}}. \end{array}$$

(*2) Intraclusters Communication.* The cluster which has selected a new cluster head needs to establish links within the cluster. If the new cluster head can directly communicate with its cluster member nodes, then the cluster head communicates with its cluster member nodes directly. Otherwise, the first CC model should be adopted, and the first cluster head node which was decided by* static clustering* is chosen as a relay node; they send messages to the node of the cluster members together. As the first cluster head nodes are able to communicate with its cluster member nodes, the new cluster head node will also be able to communicate with the cluster member nodes.

(*3) Interclusters Communication*. The cluster which has selected a new cluster head needs to establish links with its neighbor cluster. If the new cluster head can directly communicate with its adjacent cluster heads, the direct communication is reached; if cluster head *v* cannot directly communicate with the neighbor cluster head *w*, but a cluster member node *v*_*m*_ of *v* can directly communicate with the cluster head *w*, then *v*_*m*_ is chosen as the help node of *v* to communicate with *w*; if neither the cluster head *v* nor its cluster member nodes can communicate with the cluster head *w*, then the CC link is built, help nodes are chosen, and the intraclusters communication in the first step should be adopted again.

(*4) Transmission Power Reassignment.* As for the cluster member node *v*_*m*_, the transmission power of member cluster heads is calculated by the following:

(12) $$\begin{array}{c} p_{v_{m}}=\{\begin{array}{cc} \tau {\left(d_{v_{m}v}\right)}^{\kappa },\left({\left(d_{vv_{m}}\right)}^{\kappa }\leq \frac{P_{\max}}{\tau }\right), & \\ \tau {\left(d_{v_{m}u}\right)}^{\kappa },\left({\left(d_{vv_{m}}\right)}^{\kappa }>\frac{P_{\max}}{\tau }\right). & \end{array} \end{array}$$

Here, *v* is the new cluster head and *u* is the first cluster head.

As for the help node *v*_*h*_, when it communicates with another cluster head node *w*, the transmission power *p*_*v*_*h*_*w*_ is calculated by the following:

(13) pvhw={τ(dvhw)κ,((dvhw)κ≤Pmax⁡τ,(dvw)κ>Pmax⁡τ),τ∑i∈v∪H(v)(diw)−κ,((dvhw)κ>Pmax⁡τ,(dvw)κ>Pmax⁡τ).

Here, *v* is the cluster head node of the cluster it belongs to and *w* is another cluster head node. In addition, help nodes, as cluster member nodes, should be able to directly communicate with its cluster head nodes of the cluster it belongs to. The final transmission power of help node *v*_*h*_ is calculated by the following:

(14) $$\begin{array}{c} p_{v_{h}}=\max\left\{\tau {\left(d_{v_{h}v}\right)}^{\kappa },\max p_{v_{h}w}\right\}. \end{array}$$

As for the new cluster head node *v*, when it communicates with another cluster head node *w*, the transmission power of *v* is calculated by the following:

(15) pvw={τ(dvw)κ,((dvw)κ≤Pmax⁡τ),τ(duw)κ,((dvw)κ>Pmax⁡τ,(dvmw)κ≤Pmax⁡τ),τ∑i∈v∪H(v)(div)−κ,((dvw)κ>Pmax⁡τ,(dvmw)κ>Pmax⁡τ).

Here, *v*_*m*_ is the neighbor node of *v*. The final transmission power of cluster head node *v* is calculated by the following:

(16) $$\begin{array}{c} p_{v}=\max\left\{\underset{v_{m}\in M\left(v\right)}{\max}d_{vv_{m}}^{2},\max p_{vw}\right\}. \end{array}$$

## 5. Simulation Results

### 5.1. Simulation Setting

According to the simulation parameters, we do two groups experiments. The simulation parameters of the first group experiment are network capacity and average transmission power. The experimental environment is the same as [5]. In this simulation, 20–150 nodes are randomly arranged in a 500 m × 500 m area. We assume that *κ* = 2, *τ* = 10^−4^, and *B* = 20 MHZ. The value of *P*_max⁡_ is 490 mW. The value of initial energy *E* is 49 J and the energy threshold is 30 J. The simulation parameter of the second group experiment is network lifetime. 10 pairs of source and destination nodes are generated randomly. Each source node transmits data packets to the destination at the rate of 2 packets/s. Packet size is 1024 bit. We adopt Floyd algorithm for routing and assume that the MAC protocol is ideal. In order to produce more reliable results, the data are generated by averaging the data from several random topologies.

### 5.2. Experimental Results

**Figure 12:**
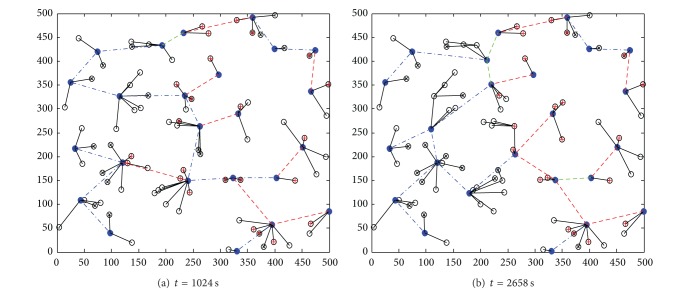
Topology evolvement by DCCPA.

**Figure 13:**
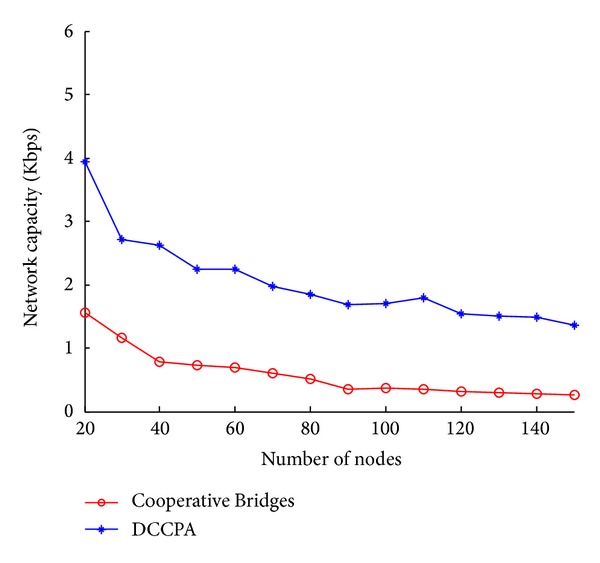
Network capacity.

**Figure 14:**
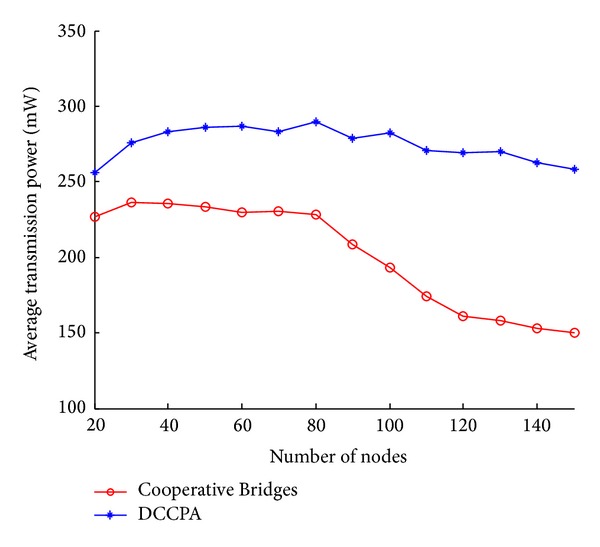
Average transmission power.

The dynamic topology evolvement by DCCPA is shown in Figure 12. At *t* = 0, as shown in Figure 10, the topology varied with the node residual energy distribution from Figures 12(a) and 12(b), the green dotted lines express the direct communication link between the cluster head, the blue dotted lines express the CC link between the cluster heads under the first CC model, and the red dotted lines express the CC link between the cluster heads under the second CC model.

(*1) The Simulation Results of the First Group Experiment.* Figure 13 shows that DCCPA's network capacity of last time increases by about 80%. DCCPA algorithm can well reduce radio interference by clustering. From Figure 14, we can see that DCCPA's average transmission power also increases. So the network capacity will be greatly improved according to formula (6).

**Figure 15:**
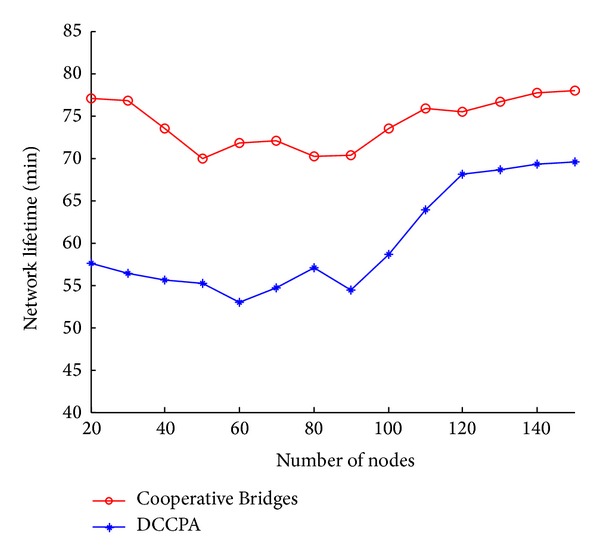
Network lifetime.

From Figure 14 we can see that the average energy consumption of the DCCPA algorithm is more than that computed by Cooperative Bridges algorithm. From Figures 10 and 11, we can see that in DCCPA there are more cluster head nodes and assistant nodes. Under normal circumstances, the transmission power of cluster head nodes and assistant nodes will be slightly higher than that of ordinary member cluster nodes which can directly communicate messages in Cooperative Bridges algorithm. Moreover, in the second step of DCCPA, cluster head rotation occurs, which means that more nodes will become cluster head nodes or assistant nodes. Therefore, the average energy consumption of DCCPA will be higher.

(*2) The Simulation Results of the Second Group Experiment.* The ultimate goal of reducing energy consumption is to extend network lifetime.  Figure 15 shows the network lifetime of DCCPA. Compared with Cooperative Bridges, the network lifetime of DCCPA is about 20% longer. According to the definition of network lifetime, it depends on the death time of the first node. The DCCPA detects that, when the residual energy of node is less than the threshold value, rotation will be adopted, in other words, reducing its transmission power while improving the transmission power of other nodes which have more residual energy. The network lifetime is lengthened through equalizing the energy consumption of nodes.

## 6. Conclusion

In this paper, we propose a dynamic cooperative clustering based power assignment (DCCPA) algorithm to solve a new topology control problem: network capacity and energy efficient in cooperative wireless ad hoc networks. To the best of our knowledge, this is the first study to investigate our proposed algorithm for solving the problem. We give a detailed description of the algorithm which aims to maximize the network capacity and network lifetime in cooperative wireless ad hoc networks. Simulation results demonstrate the high performance of DCCPA which can improve 80% network capacity and prolong 20% network lifetime compared with the Cooperative Bridges algorithm.
